# Proton pump inhibitors reduce the survival of advanced lung cancer patients with therapy of gefitinib or erlotinib

**DOI:** 10.1038/s41598-022-10938-x

**Published:** 2022-04-29

**Authors:** Chia-Han Lee, Mei-Chiou Shen, Ming-Ju Tsai, Jung-San Chang, Yaw-Bin Huang, Yi-Hsin Yang, Kun-Pin Hsieh

**Affiliations:** 1 School of Pharmacy, College of Pharmacy, Kaohsiung Medical University, 100, Shih-Chuan 1st Road, Kaohsiung, 807 Taiwan; 2 Department of Pharmacy, Kaohsiung Medical University Hospital, Kaohsiung Medical University, Kaohsiung, Taiwan; 3 Division of Pulmonary and Critical Care Medicine, Department of Internal Medicine, Kaohsiung Medical University Hospital, Kaohsiung Medical University, Kaohsiung, Taiwan; 4 Department of Internal Medicine, School of Medicine, College of Medicine, Kaohsiung Medical University, Kaohsiung, Taiwan; 5 Doctoral Degree Program in Toxicology, College of Pharmacy, Kaohsiung Medical University, Kaohsiung, Taiwan; 6 Division of Gastroenterology, Department of Internal Medicine, Kaohsiung Medical University Hospital, Kaohsiung Medical University, Kaohsiung, Taiwan; 7 National Institute of Cancer Research, National Health Research Institutes, Tainan, Taiwan

**Keywords:** Lung cancer, Oncology

## Abstract

Gefitinib and erlotinib are the first-line tyrosine kinase inhibitors (TKI) for advanced non-small-cell lung cancer. However, co-administration of either drug with proton pump inhibitors (PPI) or histamine-2 receptor antagonists (H2RA) may reduce TKI’s bioavailability. Therefore, we aimed to investigate the effects of these drug–drug interactions. We surveyed nationwide population-based databases between Jan 1, 2010, and Dec 30, 2018. Newly diagnosed patients with advanced lung adenocarcinoma who received first-line gefitinib or erlotinib were identified. Effects on overall survival (OS) and time to next treatment (TTNT) association between PPIs or H2RAs and co-administrated gefitinib or erlotinib were evaluated. PPIs or H2RAs users were defined if the period overlapped with TKIs by ≥ 20%. A total of 4340 gefitinib and 1635 erlotinib users were included. PPI group had the shortest median OS and TTNT compared to the H2RA and non-user groups (in gefitinib cohort: OS: 14.35 vs. 17.67 vs. 21.87 months; *P* < 0.0001, TTNT: 8.47 vs. 10.78 vs. 10.33 months; *P* < 0.0001); (in erlotinib cohort: OS: 16.97 vs. 20.07 vs. 23.92 months; *P* < 0.0001, TTNT: 9.06 vs. 11.85 vs. 10.90 months; *P* = 0.0808). Compared with the non-user group, the adjusted hazard ratio (aHR) of the PPI group in the gefitinib was 1.58 on OS (95% CI 1.42–1.76), 1.37 on TTNT (95% CI 1.24–1.52); in the erlotinib was 1.54 on OS (95% CI 1.30–1.82) and 1.19 on TTNT (95% CI 1.01–1.39). Concurrent use of PPIs with first-line gefitinib or erlotinib therapy was associated with a worse OS and TTNT in patients with lung adenocarcinoma harboring *EGFR* mutations.

## Introduction

Lung cancer is the leading cause of cancer-related death worldwide and in Taiwan^1–3^. The development of epidermal growth factor receptor (EGFR) tyrosine kinase inhibitors (TKIs) stimulated advances in the therapy of advanced non-small-cell lung cancer (NSCLC)^4^. When compared to traditional chemotherapy, EGFR-TKIs have superior efficacy and a more acceptable adverse effect profile, especially in treatment-naive patients^5–8^. Therefore, gefitinib, erlotinib, and afatinib have been recommended as standard therapy for first-line NSCLC treatment^9^. There are some predictors for EGFR-TKI treatment response, including adenocarcinoma, never having smoked, and *EGFR* mutation-positive status^1,3,4,10–15^. Among these factors, *EGFR* mutations are more prevalent in Asians (range 20–76%)^16^, especially in Taiwan (50–60%)^17^. Therefore, it is key to evaluate the efficacy of EGFR-TKIs in Taiwan.

The desirable efficacy of gefitinib and erlotinib could be affected by drug–drug interactions regarding their pharmacokinetic properties, such as pH-dependent solubility by oral administration route. The concurrent use of acid-suppression agents (AS), such as proton pump inhibitors (PPI) and histamine-2 receptor inhibitors (H2RA), would create a more alkaline environment in the gastrointestinal tract and lower the solubility of some EGFR-TKIs, thus influencing the drug exposure in the body^18^. Studies indicated that about 22% of patients receive PPIs and erlotinib, and 24% use PPIs concurrently with first-line gefitinib^19,20^. Due to the high frequency of prescribing PPIs and H2RAs in Taiwan, patients may receive PPIs or H2RAs high during TKI treatment^12,14^. Regarding the degree of concentration alteration, the concurrent use of ranitidine and sodium bicarbonate decreases the gefitinib area under the concentration–time curve (AUC) by 44%^21^. In healthy volunteers, when erlotinib co-administered with omeprazole, erlotinib’s maximum serum concentration (C_max_) and AUC decreased by 61% and 46%, respectively^22^. Among commonly used TKIs, the solubility of afatinib was not altered by this interaction^15^. Based on pharmacokinetic studies, the manufacturers’ suggested avoiding the intake of PPIs or H2RAs with gefitinib or erlotinib, if possible.

Several studies found controversial AS concurrent with TKIs and TKIs alone regarding clinical outcomes^19,20,23–28^. However, recently, a pooled analysis in a systematic review showed concomitant AS would reduce survival^13^. Theoretically and from a pharmacology perspective, clinical outcomes would be worse in patients receiving PPIs than H2RAs, because the ability of PPIs to inhibit gastric acid secretion is stronger than that of H2RA^29^. However, to date, no studies have supported this assumption. Therefore, the objective of this retrospective population-based cohort study was to compare how the effectiveness of first-line gefitinib or erlotinib is affected by PPIs and H2RAs. We aimed to provide suggestions on which AS to choose for patients requiring PPIs or H2RAs.

## Methods

### Data source

This retrospective cohort study used the Taiwan Cancer Registry (TCR), NHI, and Death Registry (DR) databases from Jan 1, 2010 to Dec 30, 2018. The NHI is a mandatory single-payer NHI system implemented in 1995. The NHI has covered over 99% of residents in recent years and provided comprehensive medical care. The NHI database contains clinical information on outpatient and inpatient claims data. The TCR database contains information on cancer diagnosis and the first primary treatment and the DR database contains death dates and multiple causes of death. All personal information is encrypted in the databases to protect patients' anonymity. This study was approved by the Institutional Review Board (IRB) of the Kaohsiung Medical University Hospital (KMUHIRB-E(I)-20200015). All methods were performed in accordance with the relevant guidelines and regulations. The requirement to obtain informed consent was waived by the IRB of the Kaohsiung Medical University Hospital.

### Patients with lung cancer identification and data collection

**Figure 1:**
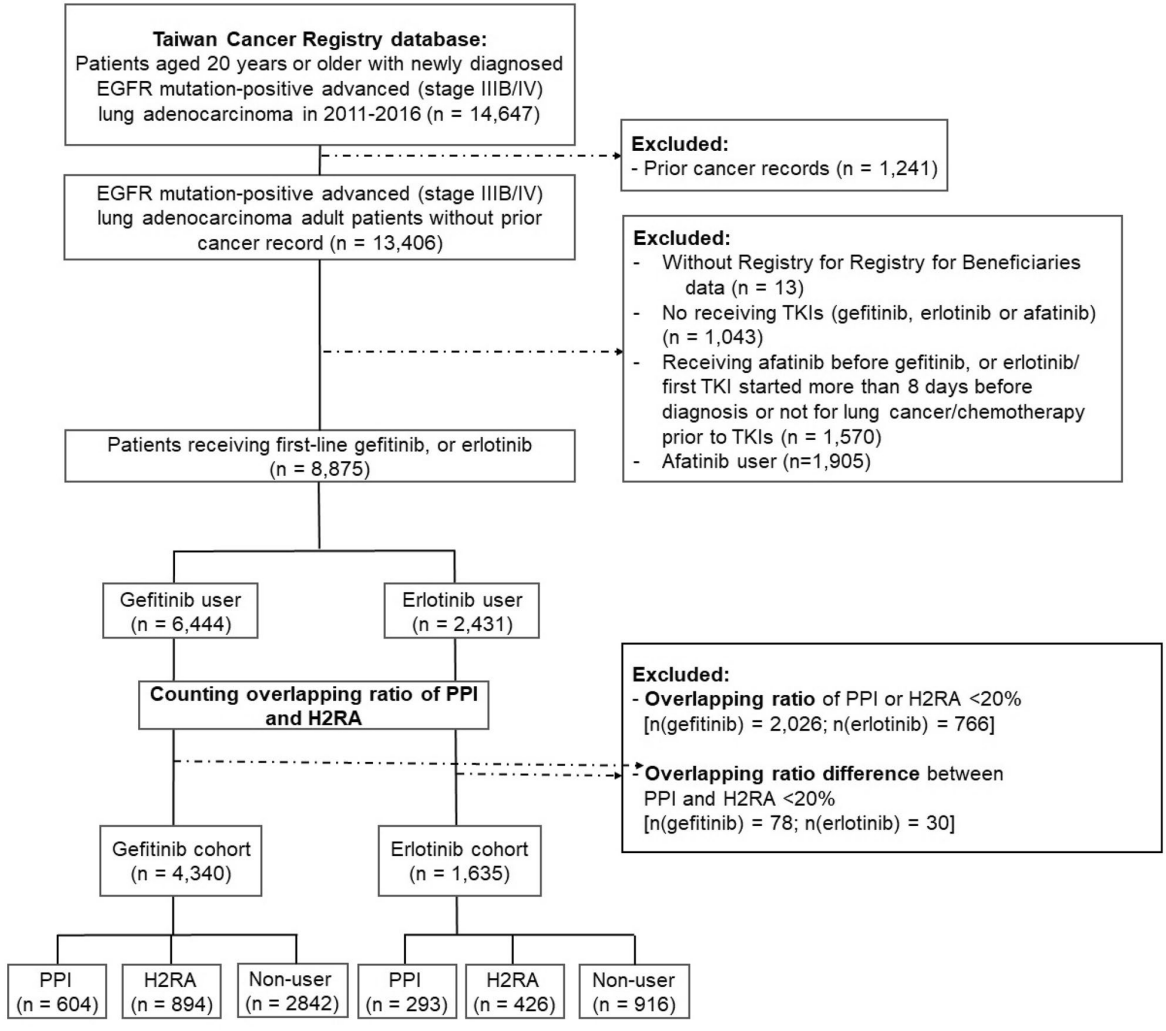
Flow chart of the study cohort.

We used ICD-O-3 code C33–C34 to identify patients with lung cancer in the TCR database from Jan 1, 2011 and Dec 31, 2016. Further inclusion criteria were: patients aged over 20 years with advanced stage (stage IIIB and IV) adenocarcinoma with *EGFR* mutations, no prior cancer record before lung cancer diagnosis, and receiving first-line treatment with gefitinib, erlotinib or afatinib. The timeline of the cohort was selected because gefitinib had been reimbursed as first-line therapy for advanced *EGFR*-mutant lung cancer since Jun 1, 2011, followed by erlotinib (since November 1, 2013). Anatomical Therapeutic Chemical (ATC) codes were used, L01EB01 (Gefitinib), L01EB02 (Erlotinib) and L01EB03 (Afatinib), to identify patients receiving EGFR-TKIs. For first-line treatment, we identified patients who had not received prior chemotherapy (Supplementary Table 3 online). The whole study population was then classified into gefitinib and erlotinib users (Fig. 1).

Patient demographics, including age, gender, stage at diagnosis, Eastern Cooperative Oncology Group (ECOG) performance status, smoking status, Charlson Comorbidity Index (CCI) score^30^, the year of diagnosis, geographic region, insurance premium, peptic ulcer disease (PUD), body surface area (BSA) and co-medications (oral steroids: betamethasone, dexamethasone, cortisone, prednisolone, triamcinolone, and methylprednisolone) were collected. The cumulative defined daily doses (cDDDs) of oral steroids during the first 3 months of receiving EGFR-TKIs were estimated per ATC/DDD Index 2022^31,32^.

### Definition of exposure to PPI and H2RA

The patient who didn’t receive any PPIs or H2RAs during EGFR-TKI therapy was defined as a non-user group. The AS agent information was identified by ATC codes (Supplementary Table 4 online). We included patients receiving PPIs or H2RAs who had overlap with the duration of TKIs ≥ 20%. The overlapping ratios were defined as the PPI or H2RA prescription duration (days) divided by the TKI treatment period (days)^19,23,24,26,27^. Patients with an overlapping ratio of PPI or H2RA with TKIs less than 20% or the difference of overlapping ratio with TKIs between PPI or H2RA less than 20% were excluded. The remaining patients in the gefitinib or erlotinib cohorts were included for final analysis and further categorized into the PPI, H2RA, and non-user groups (Fig. 1).

### Outcome measurements

To compute the follow-up time, the index date was defined as the date of TKI treatment initiation. The endpoints in clinical effectiveness were overall survival (OS) and time to the next treatment (TTNT). The OS was defined as the time from the index date to the date of death or the last day covered in the DR database (December 31, 2018). The TTNT was defined as the time from the index date to the initiation of the next systemic treatment for lung cancer, discontinuation of TKIs or central nervous system metastasis (ICD-9-CM code: 198.3, ICD-10-CM code: C79.31), the date of death, or the last day on the database (December 31, 2018), whichever happened first. Under the NHI policy, TKIs can be used until disease progression; therefore, we regarded discontinuation of TKI as a progression event.

### Statistical analysis

Descriptive analyses were used for categorical variables (percentage) and continuous variables (mean ± standard deviation). Two-sample t-tests and Chi-square tests were conducted to compare continuous and categorical variables among the two groups. The OS and TTNT analyses were performed using the Kaplan–Meier estimates and the log-rank test. Data were censored if a patient was not annotated with an event [death (for OS) or the initiation of the next treatment (for TTNT)] at the end of the database. A Cox proportional hazards model was used to evaluate independent factors that affected outcomes, and the results are reported as crude and adjusted hazard ratio (aHR) with a 95% confidence interval (95% CI). The adjusting variables included were gender, age, year of diagnosis, disease stage, ECOG performance status, CCI score, insurance premium, geographic region, PUD, BSA and the cDDDs of oral steroids. Missing data were considered as a missing category in our analyses.

For subgroup analyses, we compared the PPI group with the H2RA group in different overlapping ratios, including 20–49%, 50–79%, and 80–100% for adjusted HR to evaluate the dose-dependent effect. We also conducted a sensitivity analysis to estimate the results by changing the required overlapping ratio to at least 30%.

All analyses were two-sided, and *P* values lower than 0.05 indicate statistically significant differences. Data management and statistical analysis were performed with SAS version 9.4 (SAS Institute Inc., Cary, NC, USA).

### Ethics declarations

This article does not contain any studies with human participants or animals performed by any of the authors. This study has been approved by the Institutional Review Board of Kaohsiung Medical University Hospital (KMUHIRB-E(I)-20200015).

### Consent to participate/consent to publish

The requirement to obtain informed consent was waived by the IRB.

## Results

### Study population

A total of 14,647 adult patients were identified with newly diagnosed *EGFR* mutation-positive stage IIIB/IV lung adenocarcinoma between Jan 2011 and Dec 2016. Among them, 8875 received first-line gefitinib or erlotinib. After excluding ineligible patients, the remaining 5975 patients constituted our study cohort, including 4340 in the gefitinib cohort and 1635 in the erlotinib cohort. The TKI cohorts were further classified into the PPI, H2RA and non-user groups, 604 (13.9%), 894 (20.6%), 2842 (65.5%) in the gefitinib cohort; 293 (17.9%), 426 (26.1), 916 (56.0%) in the erlotinib cohort, respectively (Fig. 1). The prevalence of concurrent AS therapy with an overlapping ratio of more than 20% in first-line TKI users was 25% (2217/8875) under our study definition.

The patient characteristics of the gefitinib and erlotinib cohorts are included in Table 1. Over 95% of the patients in our studies were stage IV, and about 60% of patients had ECOG PS of 0–1. Non-smokers and CCI score equal to zero were the majority in our cohort. The H2RA group had a significantly higher percentage of females and the highest cDDD of oral steroids during the first 3 months of TKIs than the PPI and non-user groups in the gefitinib and erlotinib cohorts. In both cohorts' PPI group, more than 40% had PUD and had a higher proportion of BSA ≥ 1.6 m^2^.

**Table 1:** Patients characteristics of gefitinib and erlotinib cohort.

Characteristic	Gefitinib cohort (n = 4340)				Erlotinib cohort (n = 1635)			
	PPI	H2RA	Non-user	*P*	PPI	H2RA	Non-user	*P*
	N (%)	N (%)	N (%)		N (%)	N (%)	N (%)	
**No. patients**	604 (13.9)	894 (20.6)	2842 (65.5)		293 (17.9)	426 (26.1)	916 (56.0)	
**Gender**								
Female	359 (59.4)	638 (71.4)	1878 (66.1)	< 0.0001	148 (50.5)	259 (60.8)	495 (54.0)	0.0143
Male	245 (40.6)	256 (28.6)	964 (33.9)		145 (49.5)	167 (39.2)	421 (46.0)	
**Age (years)**								
Mean (SD)	68.7 (12.4)	68.9 (12.5)	66.3 (12.9)	< 0.0001	68.1 (10.8)	66.7 (11.5)	63.9 (12.3)	< 0.0001
20–65	226 (37.4)	338 (37.8)	1290 (45.4)	< 0.0001	111 (37.9)	190 (44.6)	492 (53.7)	< 0.0001
≥ 65	378 (62.6)	556 (62.2)	1552 (54.6)		182 (62.1)	236 (55.4)	424 (46.3)	
**Stage**								
IIIB	28 (4.6)	42 (4.7)	108 (3.8)	0.3861	6 (2.0)	15 (3.5)	34 (3.7)	0.3801
IV	576 (95.4)	852 (95.3)	2734 (96.2)		287 (98.0)	411 (96.5)	882 (96.3)	
**ECOG PS**								
0–1	363 (60.1)	548 (61.3)	2008 (70.7)	< 0.0001	205 (70.0)	289 (67.8)	695 (75.9)	0.0032
2	112 (18.5)	159 (17.8)	365 (12.8)		36 (12.3)	72 (16.9)	109 (11.9)	
> 2	80 (13.3)	126 (14.1)	216 (7.6)		28 (9.6)	42 (9.9)	52 (5.7)	
**Smoking status**								
Non-smokers	431 (71.4)	680 (76.1)	2213 (77.9)	0.0104	205 (70.0)	307 (72.1)	631 (68.9)	0.8344
Smokers	128 (21.2)	166 (18.6)	488 (17.2)		69 (23.6)	92 (21.6)	220 (24.0)	
**CCI score**								
0	275 (45.5)	429 (48.0)	1749 (61.5)	< 0.0001	140 (47.8)	215 (50.5)	586 (64.0)	< 0.0001
1	173 (28.7)	250 (28.0)	690 (24.3)		75 (25.6)	117 (27.5)	203 (22.2)	
≥ 2	156 (25.8)	215 (24.1)	403 (14.2)		78 (26.6)	94 (22.1)	127 (13.9)	
**Insurance Income ranks**								
No income	216 (35.8)	352 (39.4)	1078 (37.93)	0.0275	106 (36.2)	153 (35.9)	297 (32.4)	0.1028
≤ 22,000 NTD	177 (29.3)	215 (24.1)	663 (23.33)		50 (17.1)	80 (18.8)	138 (15.1)	
< 22,000 NTD	211 (34.9)	327 (36.6)	1101 (38.74)		137 (46.8)	193 (45.3)	481 (52.5)	
**PUD (%)**	281 (46.5)	198 (22.2)	99 (3.5)	< 0.0001	122 (41.6)	77 (18.1)	28 (3.1)	< 0.0001
**BSA (m**^**2**^**)**								
Medain (Q1, Q3)	1.6 (1.5, 1.7)	1.6 (1.5, 1.7)	1.6 (1.5, 1.7)		1.6 (1.5, 1.7)	1.6 (1.5, 1.7)	1.6 (1.5, 1.8)	
≥ 1.6	257 (42.6)	306 (34.2)	1120 (39.4)	0.0179	151 (51.5)	180 (42.3)	442 (48.3)	0.1021
< 1.6	293 (48.5)	499 (55.8)	1457 (51.3)		122 (41.6)	202 (47.4)	396 (43.2)	
**Sterood user (%)**	241 (39.9)	324 (36.2)	631 (22.2)		129 (44.0)	189 (44.4)	257 (28.1)	
**Steroid cDDD**								
Mean (SD)	49.1 (53.7)	50.6 (59.1)	44.3 (55.4)	0.208	58.6 (58.9)	67.2 (76.9)	40 (44.9)	< 0.0001

There were missing data in ECOG PS (gefitinib: n = 363; erlotinib: n = 107), Smoking status (gefitinib: n = 234; erlotinib: n = 111) and BSA (gefitinib: n = 408; erlotinib: n = 142).

*PPI* proton pump inhibitor, *H2RA* histamine-2 receptor antagonist, *ECOG PS* Eastern Cooperative Oncology Group performance status, *CCI* charlson comorbidity index, *NTD* new Taiwan dollar, *BSA* body surface area, *PUD* peptic ulcer disease, *cDDD* cumulative defined daily doses.

### Effect of different types of AS

**Figure 2:**
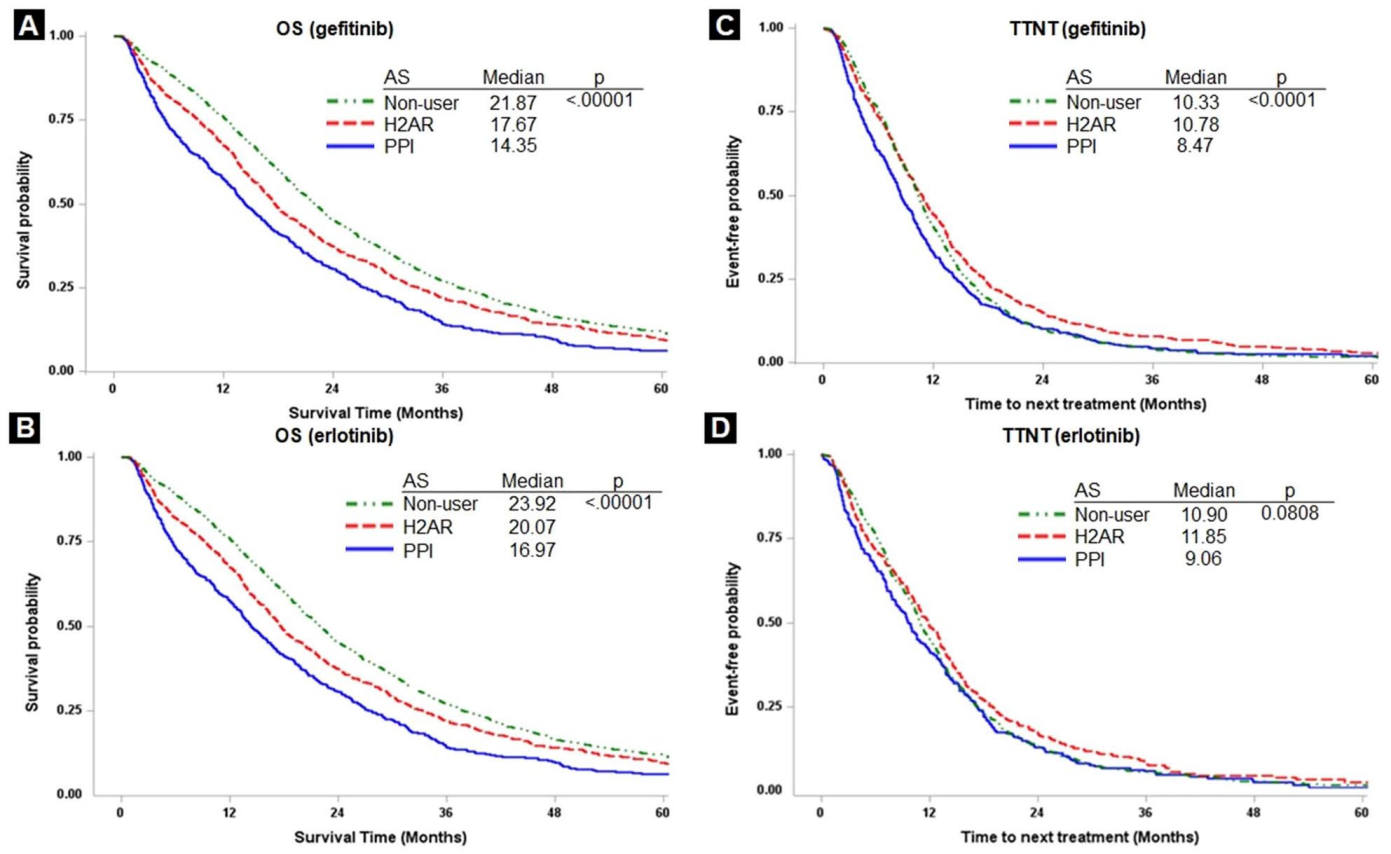
Kaplan–Meier survival curves of overall survival (OS) between PPI and H2RA groups in gefitinib (**A**) and erlotinib (**B**); and time to next treatment (TTNT) between PPI and H2RA groups in gefitinib (**C**) and erlotinib (**D**).

The median OS of the gefitinib cohort in the PPI group was significantly lower than that in the H2RA and no-user groups (14.35 vs. 17.67 vs. 21.87 months; *P* < 0.0001; Fig. 2A), and so was that of the erlotinib cohort (16.97 vs. 20.07 vs. 23.92 months; *P* < 0.0001; Fig. 2B). The median TTNT for PPI users was significantly lower than that in the H2RA group and no-user groups in the gefitinib cohort (8.47 vs. 10.78 vs. 10.33 months; *P* < 0.0001; Fig. 2C) and the erlotinib cohort (9.06 vs. 11.85 vs. 10.90 months; *P* = 0.0808; Fig. 2D).

Results of univariate and multivariable analyses of gefitinib and erlotinib are presented in Table 2. In the univariate analysis, patients receiving PPI had a significantly higher risk of death than the H2RA and the non-user groups in the two TKI cohorts. Using concurrent PPI still had a significantly negative impact on OS compared with non-user in the multivariable analysis of gefitinib (aHR = 1.58; 95% CI 1.42–1.76) and erlotinib (aHR = 1.54; 95% CI 1.30–1.82). However, concurrent H2RA had a significantly negative impact on OS compared with non-user in the gefitinib cohort (aHR = 1.14; 95% CI 1.05–1.24), but not in the erlotinib cohort (aHR = 1.00; 95% CI 0.87–1.15). Patients receiving PPI had a significantly higher risk of using the next treatment than non-users in both cohorts (gefitinib: aHR = 1.37; 95% CI 1.24–1.52; erlotinib: aHR = 1.19; 95% CI 1.01–1.39). Compared to non-user, the H2RA group in both cohorts did not have the negative impact on TTNT (gefitinib: aHR = 0.92; 95% CI 0.85–1.00; erlotinib: aHR = 0.83; 95% CI 0.73–0.94).

**Table 2:** Primary analysis and sensitivity analysis of overall survival (OS) and time to next treatment (TTNT) in the gefitinib and erlotinib cohorts.

	Gefitinib				Erlotinib			
	Univariate analysis		Multivariable analysis		Univariate analysis		Multivariable analysis	
	HR (95% CI)	*P* value	HR (95% CI)	*P*	HR (95% CI)	*P*	HR (95% CI)	*P*
**Primary analysis**								
*OS*								
Non-user	1.00		1.00		1.00		1.00	
H2RA	1.19 (1.10–1.29)	< 0.0001	1.14 (1.05–1.24)	0.003	1.16 (1.02–1.32)	0.027	1.00 (0.87–1.15)	0.992
PPI	1.50 (1.37–1.65)	< 0.0001	1.58 (1.42–1.76)	< 0.0001	1.53 (1.32–1.76)	< 0.0001	1.54 (1.30–1.82)	< 0.0001
*TTNT*								
Non-user	1.00		1.00		1.00			
H2RA	0.88 (0.82–0.95)	0.001	0.92 (0.85–1.00)	0.049	0.91 (0.81–1.02)	0.110	0.83 (0.73–0.94)	0.004
PPI	1.13 (1.04–1.24)	0.006	1.37 (1.24–1.52)	< 0.0001	1.08 (0.94–1.23)	0.288	1.19 (1.01–1.39)	0.033
**Sensitivity analysis**								
*OS*								
Non-user			1.00				1.00	
H2RA			1.16 (1.06–1.27)	0.002			0.96 (0.83–1.12)	0.613
PPI			1.65 (1.47–1.86)	< 0.0001			1.56 (1.30–1.88)	< 0.0001
*TTNT*								
Non-user			1.00				1.00	
H2RA			0.94 (0.86–1.02)	0.135			0.80 (0.70–0.92)	0.002
PPI			1.53 (1.37–1.72)	< 0.0001			1.19 (1.00–1.42)	0.049

Among other factors in both cohorts, a shortened OS was significantly associated with the male sex (except for the erlotinib cohort), older age, stage IV, ECOG PS of ≧ 2, smokers (except for the gefitinib cohort), CCI score ≧ 2 (except for the erlotinib cohort) in multivariable analyses. The male sex (except for the erlotinib cohort), stage IV, ECOG PS of ≧ 2, smokers (except for the gefitinib cohort) were associated with a poorer TTNT in both cohorts. PUD history and BSA ≥ 1.6 m^2^ had a significant positive impact on OS and TTNT. And every cDDD of oral steroid increasing increased 1% risk of OS and TTNT(Supplementary Table 1 online and Supplementary Table 2 online).

### Sensitivity analysis

When changing the definition of the required overlapping ratio from at least 20% to at least 30%, concurrent PPI had a worse OS and TTNT than did concurrent H2RA while compared to non-user in the multivariable analysis of the gefitinib cohort (OS: aHR = 1.65 vs. 1.16; TTNT: aHR = 1.53 vs. 0.94; Table 2) and the erlotinib cohort (OS: aHR = 1.56 vs. 0.96; TTNT: aHR = 1.19 vs. 0.80; Table 2).

### Subgroup analysis

**Figure 3:**
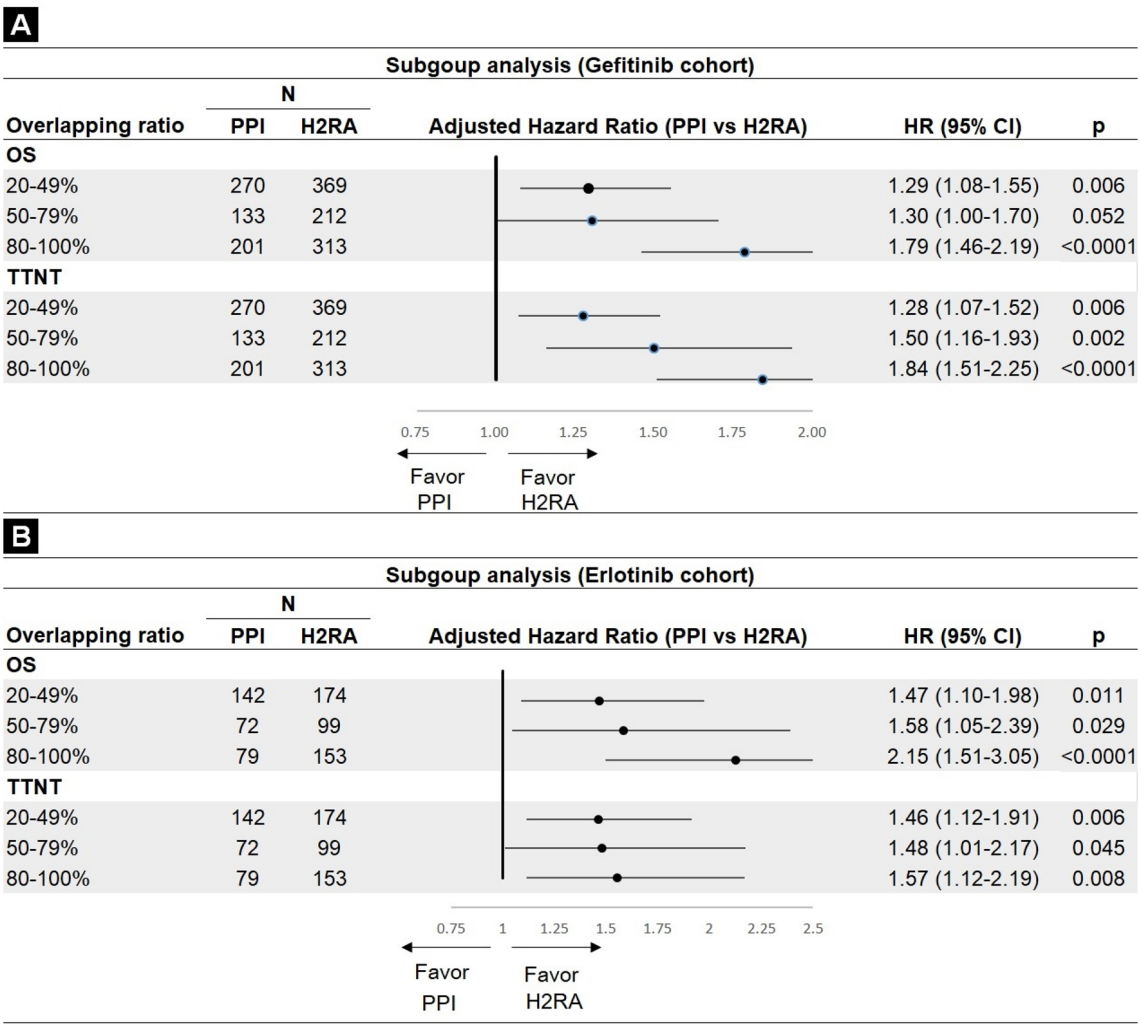
Subgroup analysis: effects of different overlapping ratios in overall survival (OS) and time to next treatment (TTNT) between PPI and H2RA groups. (**A**) gefitinib cohort and (**B**) erlotinib cohort.

For using PPIs during the TKI period, different overlapping ratios were associated with an increased mortality risk, from 20 to 49% (aHR = 1.29; 95% CI 1.08–1.55; *P* = 0.006) to 80–100% (aHR = 1.79; 95% CI 1.46–2.19; *P* < 0.0001) in the gefitinib cohort (Fig. 3A). Values in the erlotinib cohort were: 20–49% (aHR = 1.47; 95% CI 1.10–1.98; *P* = 0.011; 80–100%: aHR = 2.15; 95% CI 1.51–3.05; *P* < 0.0001)(Fig. 3B). A similar trend for TTNT was also found in both cohorts.

## Discussion

Despite manufacturers’ recommendations to avoid gefitinib or erlotinib with PPIs or H2RAs, 25% of patients with EGFR-mutant advanced lung adenocarcinoma were administered AS concurrent with first-line TKIs. This nationwide population-based cohort study demonstrated that concurrent PPI with gefitinib or erlotinib was associated with significantly poorer OS and TTNT than the H2RA and non-user. Other studies comparing the PPI and H2RA groups showed no significant differences in OS or progression-free survival (PFS) between the two groups^23,24,27^. These differing results could be explained by their smaller sample size or different study objectives. They compared concurrent AS users with non-users and did not distinguish PPI and H2RA by setting a clear definition. Furthermore, they only presented the median time and analyzed the effect of two groups in the univariate analysis without adjusting other variables to minimize potential confounders. Another possible reason could be the different inclusion criteria. Chu et al. and Sedano et al. included patients with other histology, not only adenocarcinoma, and not only first-line TKI users^24,27^.

The sensitivity analysis results were similar to those of the primary analysis in the gefitinib and erlotinib cohorts. The definition of primary analysis with an overlapping ratio of 20% was sufficient to detect the difference between the PPI and H2RA groups. The inconspicuous impact on OS in the primary analysis may be due to a relatively low required overlapping ratio. Therefore, the relationship between the two groups might still exist when overlapping ratios of PPI or H2RA were sufficient to detect differences. We could find some signs in the study conducted by Mir et al. showing that co-administration of AS and pazopanib have a negative impact on PFS and OS, while no such association was observed in the placebo cohort^33^. They chose > 80% overlap of TKI duration as the definition of AS users and concluded that a higher overlapping ratio allowed the composition of a more homogeneous group of AS users, resulting in a higher possibility of drug–drug interactions with TKI. Our study could explain the mechanism by increasing the required overlapping ratio. The difference in the degree of concentration alteration caused by the two groups might increase, resulting in a higher chance of observing the relationship between PPI and H2RA on clinical outcomes. To conclude, we found a more apparent effect of OS and TTNT in the PPI group in the sensitivity analysis when the overlapping ratio with TKIs increased to ≥ 30%.

For the subgroup analysis, supportive dose–response effects were observed when the risk of higher mortality and TTNT in gefitinib and erlotinib users receiving concurrent PPIs increased with an increasing overlapping ratio of PPI and H2RA. This supported our inference that the difference between PPI and H2RA was more likely to exist in the gefitinib than in the erlotinib cohort.

To the best of our knowledge, our study is the first comparing patients with different concurrent AS and non-user with a clear definition distinguishing them. Besides, we used nationwide population-based databases to include sufficient sample sizes. There were several limitations to this study. Firstly, we used the TTNT as a surrogate endpoint of PFS because data needed to check progressions, such as imaging tests or laboratory data, was not available. However, according to the Taiwan National Health Insurance (NHI) regulation, EGFR-TKIs should be discontinued if the disease progresses. Therefore, the outcome TTNT may be suitable for measuring disease progression in our study setting. Secondly, we could not determine the drugs prescribed without the NHI payment. In Taiwan, PPIs are only reimbursed for patients who underwent endoscopy with a diagnosis of peptic ulcer or reflux esophagitis. Therefore, we may have underestimated the overlapping ratio of PPIs and H2RAs if patients refused to undergo an invasive test or for other indications (such as preventing steroid-induced ulceration) and might purchase AS out of pocket. Thirdly, we did not analyze other drug–drug interactions with TKIs. Some other drugs interact with gefitinib or erlotinib, including CYP3A4 inhibitors or inducers. The results may be affected in different directions, and it isn't easy to quantify the effect of every drug class. However, we added co-medications (oral steroids)^31,32^ and BSA^34–37^ to adjust our analysis since these factors might affect outcomes. Lastly, osimertinib, the current standard first-line treatment of *EGFR* mutation-positive advanced NSCLC, was not included in this study because Taiwan NHI did not cover osimertinib as the first-line treatment until April 2020. The package insert indicates that osimertinib has no obvious drug-drug interaction with PPIs or H2AR. Nevertheless, our study result remains useful for clinicians to choose adequate regimens while osimertinib is unavailable.

## Conclusion

This large population-based cohort study is one of the first to demonstrate a different effect of PPI and H2RA. PPI use reduced OS and TTNT among patients with *EGFR* mutation-positive lung adenocarcinoma who received first-line gefitinib or erlotinib compared with those under H2RA or non-AS users. A dose–response effect was also found when increasing the overlapping ratio definition. The following recommendations are mainly for patients requiring AS during their TKI period. For patients already receiving gefitinib or erlotinib, H2RAs should be prescribed.

## Supplementary Information

Supplementary Tables.

## Data Availability

The data supporting the results of this study can be found in the supporting materials.
