# Supplementary material for: Proton pump inhibitors reduce the survival of advanced lung cancer patients with therapy of gefitinib or erlotinib
Source: Sci Rep. 2022 Apr 29;12:7002. doi: 10.1038/s41598-022-10938-x (PMC9054789; doi:10.1038/s41598-022-10938-x)
Supplement: Supplementary file 1 — Supplementary Tables. [file 41598_2022_10938_MOESM1_ESM.pdf]

Proton Pump Inhibitors Reduce the Survival of Advanced Lung Cancer Patients with Therapy of Gefitinib or Erlotinib

Chia-Han Lee, Mei-Chiou Shen, Ming-Ju Tsai, Jung San Chang, Yaw-Bin Huang, Yi-Hsin Yang, Kun-Pin Hsieh*

Supplementary Table 1 Primary analysis and sensitivity analysis of overall survival (OS) and time to next treatment (TTNT) in the gefitinib cohort

	Primary analysis						Sensitivity analysis		
	Univariate analysis			Multivariable analysis			Multivariable analysis		
Variable	HR	95% CI	p	HR	95% CI	p	HR	95% CI	p
OS									
Non-user	1.00			1.00			1.00		
H2RA	1.19	(1.10– 1.29)	<0.0001	1.14	(1.05– 1.24)	0.003	1.16	(1.06– 1.27)	0.002
PPI	1.50	(1.37– 1.65)	<0.0001	1.58	(1.42– 1.76)	<0.0001	1.65	(1.47– 1.86)	<0.0001
Female	1.00			1.00			1.00		
Male sex	1.26	(1.18– 1.35)	<0.0001	1.62	(1.30– 2.01)	<0.0001	1.63	(1.31– 2.04)	<0.0001
Age 20–65	1.00			1.00			1.00		
Age ≧65	1.41	(1.32– 1.50)	<0.0001	2.42	(1.92– 3.06)	<0.0001	2.51	(1.97– 3.20)	<0.0001
Stage IIIB	1.00			1.00			1.00		
Stage IV	1.73	(1.45– 2.07)	<0.0001	1.70	(1.42– 2.03)	<0.0001	1.67	(1.38– 2.01)	<0.0001
ECOG 0–1	1.00			1.00			1.00		
ECOG 2	1.93	(1.77– 2.11)	<0.0001	1.75	(1.60– 1.92)	<0.0001	1.74	(1.58– 1.91)	<0.0001
ECOG >2	3.20	(2.88– 3.56)	<0.0001	4.60	(3.51– 6.03)	<0.0001	4.37	(3.30– 5.80)	<0.0001
Non-smokers	1.00			1.00			1.00		
Smokers	1.25	(1.15– 1.35)	<0.0001	1.07	(0.97– 1.19)	0.158	1.08	(0.97– 1.20)	0.142
CCI score 0	1.00			1.00			1.00		
CCI score 1	1.11	(1.03– 1.20)	0.006	0.99	(0.91– 1.07)	0.733	0.99	(0.91– 1.07)	0.774
CCI score ≧ 2	1.45	(1.33– 1.58)	<0.0001	1.22	(1.12– 1.34)	<0.0001	1.22	(1.11– 1.34)	<0.0001
PUD	0.92	(0.83– 1.01)	0.066	0.56	(0.41– 0.76)	0.0003	0.58	(0.42– 0.81)	0.001
BSA <1.6	1.00			1.00			1.00		
BSA ≥1.6	0.89	(0.83– 0.95)	0.001	0.81	(0.75– 0.87)	<0.0001	0.80	(0.74– 0.87)	<0.0001
Steroids DDD	1.01	(1.01– 1.01)	<0.0001	1.00	(1.00– 1.01)	0.0001	1.00	(1.00– 1.01)	0.0003
TTNT									
Non-user	1.00			1.00			1.00		
H2RA	0.88	(0.82– 0.95)	0.001	0.92	(0.85– 1.00)	0.049	0.94	(0.86– 1.02)	0.135
PPI	1.13	(1.04– 1.24)	0.006	1.37	(1.24– 1.52)	<0.0001	1.53	(1.37– 1.72)	<0.0001
Female	1.00			1.00			1.00		
Male sex	1.20	(1.12– 1.28)	<0.0001	1.25	(1.15– 1.36)	<0.0001	1.23	(1.13– 1.34)	<0.0001
Age 20–65	1.00			1.00			1.00		
Age ≧65	1.00	(0.94– 1.06)	1.000	0.91	(0.85– 0.97)	0.006	0.91	(0.85– 0.97)	0.007
Stage IIIB	1.00			1.00			1.00		
Stage IV	1.78	(1.52– 2.09)	<0.0001	1.66	(1.41– 1.95)	<0.0001	1.65	(1.39– 1.96)	<0.0001
ECOG 0–1	1.00			1.00			1.00		
ECOG 2	1.39	(1.28– 1.52)	<0.0001	1.38	(1.27– 1.51)	<0.0001	1.38	(1.26– 1.52)	<0.0001
ECOG >2	1.90	(1.71– 2.10)	<0.0001	3.17	(2.49– 4.03)	<0.0001	3.03	(2.35– 3.90)	<0.0001

Non-smokers	1.00			1.00			1.00		
Smokers	1.13	(1.04– 1.22)	0.003	0.98	(0.89– 1.08)	0.717	0.99	(0.89– 1.09)	0.785
CCI score 0	1.00			1.00			1.00		
CCI score 1	0.98	(0.91– 1.05)	0.488	0.97	(0.90– 1.04)	0.343	0.95	(0.88– 1.03)	0.225
CCI score ≧ 2	1.05	(0.97– 1.14)	0.208	1.07	(0.99– 1.17)	0.106	1.09	(0.99– 1.19)	0.075
PUD	0.71	(0.65– 0.78)	<0.0001	0.45	(0.33– 0.60)	<0.0001	0.47	(0.34– 0.64)	<0.0001
BSA <1.6	1.00			1.00			1.00		
BSA ≥1.6	0.95	(0.89– 1.01)	0.083	0.87	(0.81– 0.93)	<0.0001	0.88	(0.81– 0.94)	0.001
Steroids cDDD	1.00	(1.00– 1.01)	<0.0001	1.01	(1.00– 1.01)	<0.0001	1.00	(1.00– 1.01)	<0.0001

Notes: 1. The hazard ratio of the ECOG missing and smoking missing categories was not shown.

2. Year of diagnosis, geographic region, and insurance income ranks were also adjusted.

Abbreviations: AS = acid-suppression agents; PPI = proton pump inhibitor; ECOG PS= Eastern Cooperative Oncology Group performance status; CCI = Charlson Comorbidity Index; BSA = Body Surface Area; PUD = peptic ulcer disease; cDDD = cumulative Defined Daily Doses

Supplementary Table 2 Primary analysis and sensitivity analysis of overall survival (OS) and time to next treatment (TTNT) in the erlotinib cohort

	Primary analysis						Sensitivity analysis		
	Univariate analysis			Multivariable analysis			Multivariable analysis		
Variable	HR	95% CI	p	HR	95% CI	p	HR	95% CI	p
OS									
Non-user	1.00			1.00			1.00		
H2RA	1.16	(1.02– 1.32)	0.027	1.00	(0.87– 1.15)	0.992	0.96	(0.83– 1.12)	0.613
PPI	1.53	(1.32– 1.76)	<0.0001	1.54	(1.31– 1.82)	<0.0001	1.56	(1.30– 1.88)	<0.0001
Female	1.00			1.00			1.00		
Male sex	1.17	(1.05– 1.30)	0.006	1.41	(1.00– 2.00)	0.051	1.40	(0.97– 2.02)	0.071
Age 20–65	1.00			1.00			1.00		
Age ≧65	1.47	(1.31– 1.64)	<0.0001	3.29	(2.24– 4.83)	<0.0001	3.27	(2.18– 4.90)	<0.0001
Stage IIIB	1.00			1.00			1.00		
Stage IV	1.49	(1.07– 2.07)	0.017	1.58	(1.13– 2.19)	0.007	1.65	(1.15– 2.35)	0.006
ECOG 0–1	1.00			1.00			1.00		
ECOG 2	2.11	(1.81– 2.46)	<0.0001	2.02	(1.72– 2.37)	<0.0001	2.02	(1.71– 2.39)	<0.0001
ECOG >2	3.08	(2.54– 3.74)	<0.0001	6.15	(3.92– 9.65)	<0.0001	6.20	(3.85– 9.96)	<0.0001
Non-smokers	1.00			1.00			1.00		
Smokers	1.14	(1.00– 1.29)	0.052	1.21	(1.04– 1.41)	0.014	1.19	(1.01– 1.40)	0.033
CCI score 0	1.00			1.00			1.00		
CCI score 1	1.09	(0.95– 1.24)	0.227	0.96	(0.83– 1.10)	0.524	0.95	(0.82– 1.10)	0.477
CCI score ≧ 2	1.36	(1.18– 1.57)	<0.0001	1.11	(0.94– 1.30)	0.211	1.09	(0.92– 1.29)	0.328
PUD	0.92	(0.79– 1.08)	0.330	0.22	(0.12– 0.40)	<0.0001	0.26	(0.14– 0.49)	<0.0001
BSA <1.6	1.00			1.00			1.00		
BSA ≥1.6	0.80	(0.72– 0.90)	0.0002	0.70	(0.61– 0.80)	<0.0001	0.72	(0.62– 0.82)	<0.0001
Steroids DDD	1.01	(1.00– 1.01)	<0.0001	1.01	(1.00– 1.01)	<0.0001	1.01	(1.00– 1.01)	<0.0001
TTNT									
Non-user	1.00			1.00			1.00		
H2RA	0.91	(0.81– 1.02)	0.110	0.83	(0.73– 0.94)	0.004	0.80	(0.70– 0.92)	0.002
PPI	1.08	(0.94– 1.23)	0.288	1.19	(1.01– 1.39)	0.033	1.19	(1.00– 1.42)	0.049
Female	1.00			1.00			1.00		
Male sex	1.07	(0.97– 1.19)	0.161	1.05	(0.93– 1.20)	0.430	1.08	(0.94– 1.24)	0.281
Age 20–65	1.00			1.00			1.00		
Age ≧65	1.06	(0.96– 1.17)	0.266	1.05	(0.94– 1.17)	0.419	1.06	(0.94– 1.19)	0.358
Stage IIIB	1.00			1.00			1.00		
Stage IV	1.36	(1.02– 1.80)	0.034	1.41	(1.06– 1.88)	0.019	1.47	(1.08– 1.99)	0.014
ECOG 0–1	1.00			1.00			1.00		
ECOG 2	1.44	(1.25– 1.68)	<0.0001	1.42	(1.22– 1.66)	<0.0001	1.43	(1.22– 1.68)	<0.0001
ECOG >2	1.92	(1.59– 2.32)	<0.0001	3.51	(2.41– 5.12)	<0.0001	3.77	(2.53– 5.60)	<0.0001

Non-smokers	1.00			1.00			1.00		
Smokers	1.16	(1.03– 1.30)	0.016	1.22	(1.06– 1.40)	0.005	1.17	(1.01– 1.36)	0.035
CCI score 0	1.00			1.00			1.00		
CCI score 1	1.08	(0.96– 1.22)	0.223	1.06	(0.93– 1.20)	0.377	1.03	(0.90– 1.18)	0.636
CCI score ≧ 2	1.08	(0.95– 1.24)	0.240	1.09	(0.94– 1.26)	0.251	1.11	(0.95– 1.29)	0.200
PUD	0.75	(0.65– 0.87)	0.0001	0.44	(0.28– 0.70)	0.001	0.54	(0.33– 0.88)	0.012
BSA <1.6	1.00			1.00			1.00		
BSA ≥1.6	0.88	(0.79– 0.98)	0.016	0.88	(0.73– 1.07)	0.212	0.83	(0.73– 0.94)	0.003
Steroids cDDD	1.00	(1.00– 1.01)	<0.0001	1.01	(1.00– 1.01)	<0.0001	1.01	(1.01– 1.01)	<0.0001

Notes: 1. The hazard ratio of the ECOG missing and smoking missing categories was not shown.

2. Year of diagnosis, geographic region, and insurance income ranks were also adjusted.

Abbreviations: AS = acid-suppression agents; PPI = proton pump inhibitor; ECOG PS= Eastern Cooperative Oncology Group performance status; CCI = Charlson Comorbidity Index; BSA = Body Surface Area; PUD = peptic ulcer disease; cDDD = cumulative Defined Daily Doses

Supplementary Table 3 ATC codes of chemotherapy

**Agent**	**ATC code**
Bevacizumab	L01XC07
Carboplatin	L01XA02
Cisplatin	L01XA01
Docetaxel	L01CD02
Etoposide	L01CB01
Gemcitabine	L01BC05
Paclitaxel	L01CD01
Pemetrexed	L01BA04
Tegafur	L01BC03
TS-1	L01BC53
Vinorelbine	L01CA04

Supplementary Table 4 ATC codes of acid-suppression agent

**Agent**	**ATC code**
PPI	
Omeprazole	A02BC01
Pantoprazole	A02BC02
Lansoprazole	A02BC03
Rabeprazole	A02BC04
Esomeprazole	A02BC05
Dexlansoprazole	A02BC06
H2RA	
Cimetidine	A02BA01
Ranitidine	A02BA02
Famotidine	A02BA03
Nizatidine	A02BA04
